# Evidence for pollinator cost and farming benefits of neonicotinoid seed coatings on oilseed rape

**DOI:** 10.1038/srep12574

**Published:** 2015-08-13

**Authors:** G. E. Budge, D. Garthwaite, A. Crowe, N. D. Boatman, K. S. Delaplane, M. A. Brown, H. H. Thygesen, S. Pietravalle

**Affiliations:** 1 Fera, Sand Hutton, York, YO41 1LZ, UK; 2 Department of Entomology, University of Georgia, Athens, GA 30602, USA; 3 Animal and Plant Health Agency, Sand Hutton, York, YO41 1LZ, UK

## Abstract

Chronic exposure to neonicotinoid insecticides has been linked to reduced survival of pollinating insects at both the individual and colony level, but so far only experimentally. Analyses of large-scale datasets to investigate the real-world links between the use of neonicotinoids and pollinator mortality are lacking. Moreover, the impacts of neonicotinoid seed coatings in reducing subsequent applications of foliar insecticide sprays and increasing crop yield are not known, despite the supposed benefits of this practice driving widespread use. Here, we combine large-scale pesticide usage and yield observations from oilseed rape with those detailing honey bee colony losses over an 11 year period, and reveal a correlation between honey bee colony losses and national-scale imidacloprid (a neonicotinoid) usage patterns across England and Wales. We also provide the first evidence that farmers who use neonicotinoid seed coatings reduce the number of subsequent applications of foliar insecticide sprays and may derive an economic return. Our results inform the societal discussion on the pollinator costs and farming benefits of prophylactic neonicotinoid usage on a mass flowering crop.

Insects underpin the seed and fruit formation of pollinator-dependent crops that make up a critical fraction of the human diet^1^,^2^. Since 1961, the global cultivated area of insect-dependent crops has trebled^3^, while at the same time several key insect pollinator groups have declined^4^,^5^. This has led to concerns that pollination deficits may limit crop production^6^. Agricultural intensification reduces the diversity of food plants upon which insects depend^7^ and increases pollinators’ exposure to multiple pesticides^8^ that may act synergistically to increase insecticidal activity^9^. Neonicotinoids are potent nerve stimulants with a high affinity for insect nicotinic acetylcholine receptors^10^ and include the N-nitroguanidine group (clothianidin, imidacloprid or thiamethoxam) used as seed coatings, and the less toxic^11^ N-cyanoamidine group (acetamiprid and thiacloprid) used as foliar insecticide sprays. Neonicotinoids are readily absorbed by plants, which transport them systemically providing pest protection throughout all plant tissues. The versatility of application and favourable pest control properties have contributed to neonicotinoids becoming the most widely used insecticides in the world^12^, with over 90% of usage in the form of seed coatings of clothianidin, imidacloprid or thiamethoxam^13^.

Oilseed rape (OSR; *Brassica napus*) is the most widely planted oilseed crop in Europe^14^ and provides a mass-flowering pollen and nectar resource highly attractive to many species of pollinator, including honey bees^15^,^16^. Neonicotinoid seed coating has become common practice on OSR across the UK in recent years^13^, offering the farmer a new class of insecticide active against important pests such as the cabbage stem flea beetle (*Psylliodes chrysocephala*) and the peach potato aphid (*Myzus persicae;* Fig. 1).

Prophylactic application of neonicotinoids as a seed coating helps to protect the crop against pests for up to 10 weeks post planting^17^, reducing the need for subsequent weather-dependent applications of foliar insecticide sprays using older chemistries such as organophosphates, pyrethroids and carbamates, to which some pests have developed resistance^18^,^19^. However, neonicotinoid residues persist in plant tissues long enough to be detectable in OSR pollen and nectar^20^,^21^, providing a potential route for mass exposure to pollinators. Indeed the N-nitroguanidine neonicotinoids have been linked experimentally to changes in pollinator foraging behaviour^22^, reduced survival of individual insects^23^,^24^, decelerated colony growth^25^,^26^ and in the case of bumble bees, colony failure^27^. Concern that neonicotinoid seed coatings may be harming pollinators has led to a two year restriction on their use for mass flowering crops across the EU, starting in December 2013^28^. However, real-world datasets that link the usage of neonicotinoid seed coatings to pollinator mortality are lacking at the agroecosystem level.

There is little doubt that pests such as cabbage stem flea beetle and the peach potato aphid and its associated viruses (Fig. 1) cause significant reductions in OSR yield in untreated crops^17^,^29^,^30^ and that neonicotinoid seed coatings provide a level of control for these pests^30^,^31^. However, applying a neonicotinoid as a seed coating costs farmers over three times more than a single foliar insecticide spraying^32^, yet the additional yield benefit of neonicotinoid seed coating — in comparison to pest control regimes that only use foliar insecticide applications — has never been quantified. Statements on the benefits of neonicotinoid usage on OSR seed assume that the protective benefits of neonicotinoid seed coatings post-planting avert the need for a farmer to apply a spray of foliar insecticides in the Autumn^32^. However, the foliar pesticide application behaviours of farmers who use neonicotinoid seed coatings have never been assessed. The honey bee (*Apis mellifera*) can be regarded as the most important commercial pollinator globally, responsible for at least 90% of commercial pollination^33^. Honey bees are the most frequent flower visitors to OSR^6^,^15^, and their pollinating activities contribute to seed pod weight and pod set^6^. Our study fuses large datasets that describe seed and foliar pesticide usage, land use, OSR yield, meteorological conditions and honey bee colony losses to provide a unique opportunity to investigate the potential costs to pollinators and benefits to farmers of neonicotinoid seed coatings on OSR at a national level.

## Results

### Exploring factors to explain honey bee colony losses

**Figure 2:**
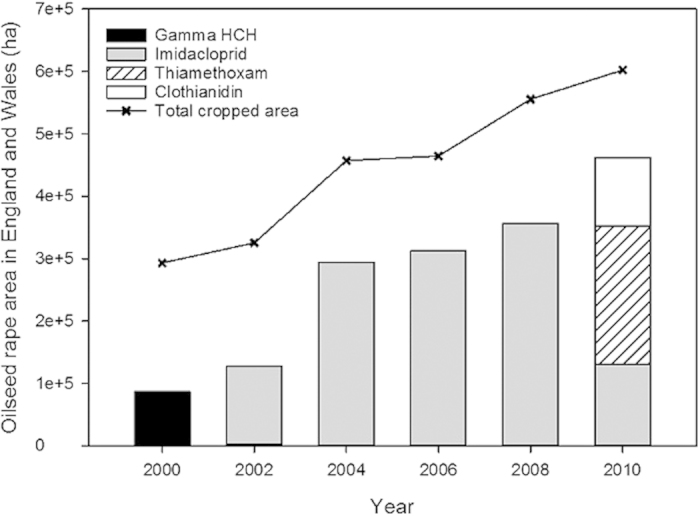
Total area of oilseed rape cropped across England and Wales (ha) including the area treated with insecticide seed coatings. More recent seed coated insecticide usage is dominated by the neonicotinoids clothianidin, imidacloprid and thiamethoxam, which replaced Gamma HCH.

Datasets that described pesticide usage, land use, OSR yield, meteorological conditions and honey bee colony losses were successfully linked for each of nine regions across England and Wales from 2000 to 2010 (see Methods). Agricultural data from the June survey showed that the total cropped area of OSR for England and Wales doubled between 2000 (293,378 ha) and 2010 (602,270 ha; Fig. 2). National pesticide usage data indicated a steady increase in the use of neonicotinoid seed coatings, which began replacing the organochlorine Gamma HCH with <1% of the planted OSR area in 2000, and increased to cover over 75% of the planted OSR area in 2010 (Fig. 2).

Pesticide usage data, from between 6,000 ha and 18,000 ha of OSR annually, were the most spatially and temporally restricted, so all datasets were considered at the same regional, biennial scale (see Methods). Two region/year combinations (West Midlands in 2000 and Wales in 2006) were excluded due to missing pesticide usage data, leaving 52 region/year combinations (Supplementary Table S1). The proportion of in-season honey bee colony losses was derived from colony health inspections across England and Wales after excluding apiaries that contained no notifiable disease (see Methods). In total, there were 126,220 colony observations, of which 10,725 honey bee colonies were found to be dead (8.5% colony losses; Supplementary Table S1).

First we used the proportion of honey bee colony losses as the response variable, and region, meteorological conditions, summed neonicotinoid usage on OSR seed (to include clothianidin, imidacloprid and thiamethoxam; kg/m^2^) and density of OSR grown (m^2^/m^2^) as the explanatory variables in a quasi-binomial generalized linear model (see Methods). The model output indicated that honey bee colony mortality differed significantly due to region, *SummerTemp*_*min*_, *SummerSun* and *SpringTemp*_*max*_ (Table 1). Neonicotinoid usage on OSR seed (*p* = 0.41) and density of OSR grown (*p* = 0.11) did not explain additional variation for honey bee colony mortality and were not added to the final model. A single value with high leverage (Wales in 2008) was removed from the model to investigate whether this point influenced the reported relationships, but the results after removal remained unchanged (data not shown).

**Figure 3:**
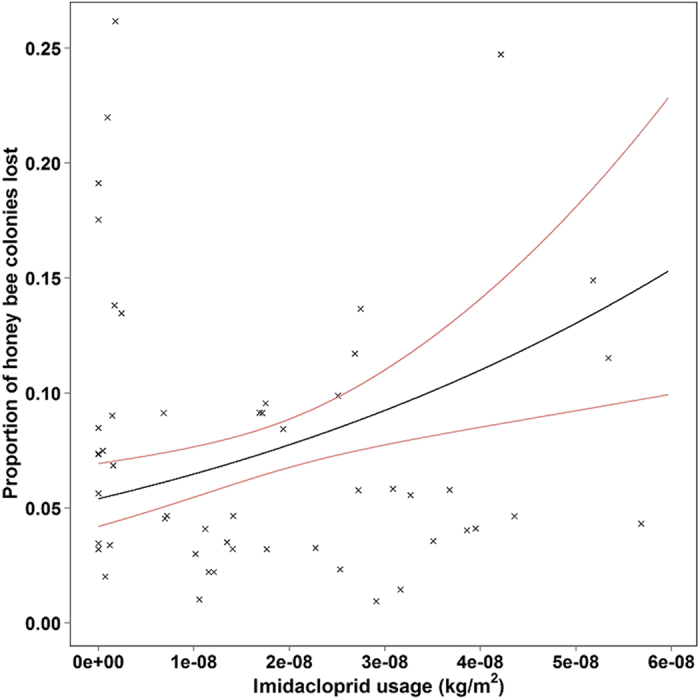
Predicted relationship between regional imidacloprid usage on oilseed rape (kg/m^2^) assessed biennially from 2000 to 2010 and the proportion of dead honey bee colonies after region had been taken into account (black line; n = 52; *p* = 0.001). Red lines represent 95% confidence intervals.

Given the dominance of imidacloprid usage as an insecticide seed coating on OSR between 2000 and 2008 (Fig. 2), we repeated the above quasi-binomial generalized linear model substituting summed neonicotinoid usage on OSR seed (kg/m^2^) with that of only imidacloprid. Region remained the strongest predictor of honey bee colony loss, however once the differences between regions had been accounted for, imidacloprid usage on OSR (kg/m^2^) had a positive relationship with honey bee colony losses such that increased regional usage was linked to higher honey bee colony losses (Table 2; Fig. 3). A single value with a high residual (East Midlands in 2008) was removed from the model, but again the results remained unchanged (data not shown). Given the lack of clothianidin and thiamethoxam usage prior to 2010 (Fig. 2), it was not possible to analyse for an individual effect of these neonicotinoid seed coatings.

We then checked for spurious results due to the possible correlation between the OSR density (m^2^/m^2^) and imidacloprid usage on OSR seed by forcing the model described in Table 2 to include OSR density before imidacloprid usage. The results indicated that honey bee colony loss was not significantly related to the density of OSR (deviance ratio = 2.43; df = 1, 41; *p* = 0.13), and the relationship between imidacloprid usage and honey bee colony losses remained significant (deviance ratio = 9.36; df = 1,41; *p* = 0.004), suggesting that our datasets contained sufficient temporal spread to decouple imidacloprid usage on OSR seed from the density of OSR grown.

### Quantifying foliar insecticide sprays

Pesticide usage survey data were used to identify the number of foliar insecticide sprays associated with winter OSR crops between 2000 and 2010 (see Methods). Over 99% of all foliar insecticide applications were pyrethroids, including Cypermethrin (47%) Lambda-cyhalothrin (19%), Alpha-cypermethrin (12%), Tau-fluvalinate (9%), Zeta-cypermethrin (8%), Deltamethrin(4%) and Bifenthrin (1%). Carbamates (pirimicarb; 0.5%), organophosphates (Chlorpyrifos and Dimethoate; 0.1%) and neonicotinoids from the N-cyanoamidine group (Thiacloprid; 0.1%) were rarely used as foliar insecticide sprays. Pesticide usage records indicated that no neonicotinoids from the N-nitroguanidine group (clothianidin, imidacloprid or thiamethoxam) had been used as foliar insecticide sprays.

**Figure 4:**
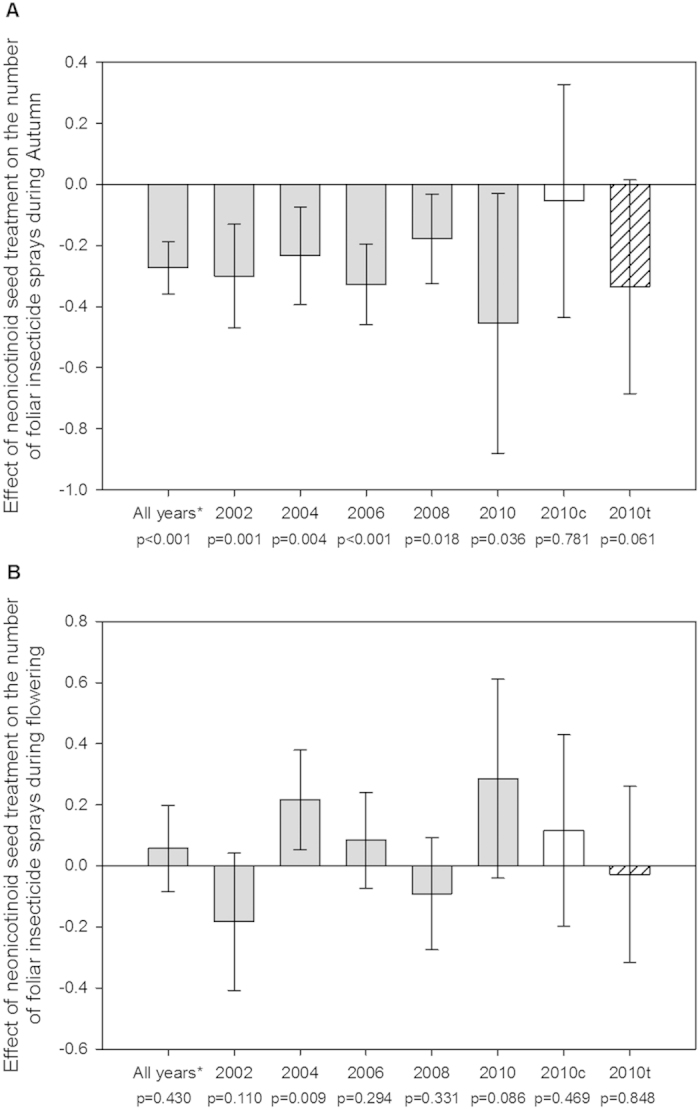
Estimated effect of imidacloprid (grey), clothianidin (clear) and thiamethoxam (hatched) seed treatments on the number of foliar insecticide sprays used during autumn (**A**) and flowering (**B**) on winter oilseed rape when compared to crops that received no insecticide seed treatment. Estimates, p-values and 95% confidence intervals are derived from Poisson models for each individual year or mixed effect Poison models for all years (imidacloprid only;*).

The number of seasonally applied foliar insecticide sprays (either autumn or during flowering) was used as the response variable in a Poisson regression model with a *log* link function with insecticide seed coating (none, clothianidin, imidacloprid or thiamethoxam) as an explanatory variable and region as a fixed effect (see Methods). Data were excluded for 2000 because of the low number of observations for imidacloprid (n = 2) and data on the spring planting of OSR were removed to focus on foliar insecticide sprays on the more common winter sown OSR. The number of foliar insecticide sprays was assessed for 4,230 fields totalling 73,051 ha of winter OSR crops across England and Wales (Table 3). Regression models predicted a consistent negative effect of imidacloprid seed treatment on the number of insecticide sprays applied in autumn in each year (Fig. 4A). This was supported by a mixed effect Poisson model combining data from across all years for imidacloprid seed treated crops (*p* < 0.001; Fig. 4A). A positive effect was noted for imidacloprid seed treatment on the number of insecticide sprays during flowering in 2004 (Fig. 4B), however a mixed effect Poisson model combining data from across all years for imidacloprid seed treated crops suggested no consistent increase in the number of foliar insecticide applications during flowering (*p* = 0.430: Fig. 4B).

### Yield benefits of neonicotinoid seed coatings

**Figure 5:**
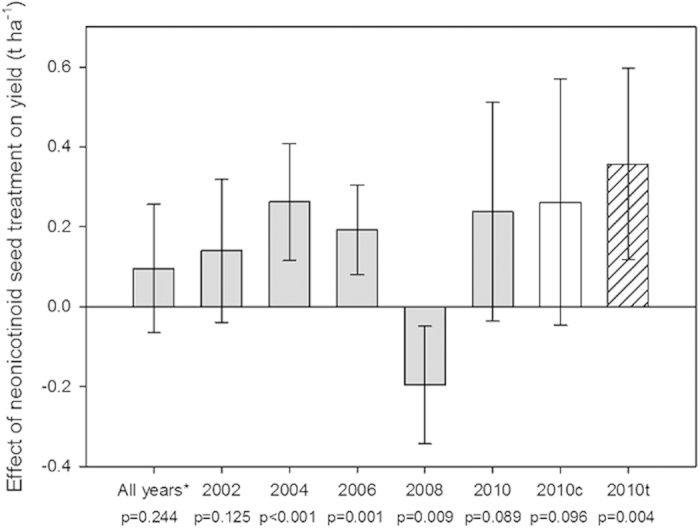
Estimated effect of imidacloprid (grey), clothianidin (clear) and thiamethoxam (hatched) seed treatments on oilseed rape yield (t ha^−1^) when compared to crops that received no insecticide seed treatment. Estimates, p-values and 95% confidence intervals are derived from linear models for each individual year or a mixed effect linear model for all years (imidacloprid only;*).

Yield (t ha^−1^) was used as the response variable in a linear regression with insecticide seed coating (none, clothianidin, imidacloprid or thiamethoxam) as an explanatory variable and region, the proportion of farm-saved seed and the proportion of hybrid seed as fixed effects (see Methods). Not all fields assessed for the number of foliar insecticide applications had associated yield data, leading to a slightly reduced dataset for yield comparisons. The yield (t ha^−1^) was assessed for 2801 fields totalling 47,120 ha of winter OSR crops across England and Wales (Table 3). Linear models predicted a positive effect of neonicotinoid seed treatment on yield (t ha^−1^) for 2004, 2006 and 2010 (thiamethoxam only; Fig. 5). However, a negative effect on yield was predicted for imidacloprid seed treated crops in 2008 when compared to crops that received no insecticide seed treatment (Fig. 5). A linear mixed model combining data from across all years found no overall effect of imidacloprid seed treatment on yield (*p* = 0.248; Fig. 5).

### Partial budget analysis of neonicotinoid seed coatings

**Figure 6:**
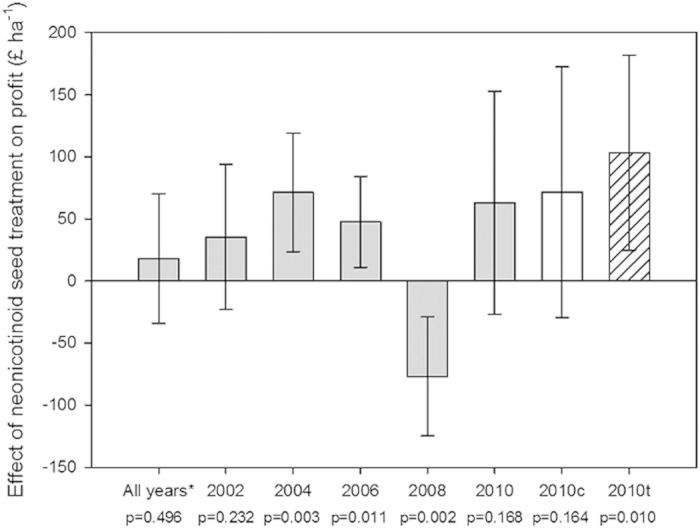
Estimated effect of imidacloprid (grey), clothianidin (clear) and thiamethoxam (hatched) seed treatments on farming profit (£ ha^−1^) when compared to crops that received no insecticide seed treatment. Estimates, p-values and 95% confidence intervals are derived from linear models for each individual year or a mixed effect linear model for all years (imidacloprid only;*).

We completed a partial budget analysis for using prophylactic neonicotinoid seed coatings by combining our new data on yield and foliar insecticide usage with industry figures on insecticide treatment costs for farmers (see Methods)^32^. The results of the budget analysis mirrored the yield, with positive of neonicotinoid seed treatment on profit (£ ha^−1^) for 2004, 2006 and 2010 (thiamethoxam only) and a negative effect in 2008 (Fig. 6). A linear mixed model combining data from across all years found no overall effect of imidacloprid seed treatment on farming profits (*p* = 0.248; Fig. 6).

## Discussion

The farming benefit of using neonicotinoid seed coatings to reduce subsequent foliar insecticide sprays and increase crop yield for OSR was not known prior to this study, despite this practice being widespread. We combined pesticide usage and yield observations from over 76,000 ha of OSR over five replicate years to provide the first evidence that farmers who used imidacloprid seed coatings consistently reduced the number of foliar insecticide sprays used in the autumn, but not during flowering (Fig. 4). Our observations fit well with the known efficacy of such seed coatings against cabbage stem flea beetle (up to 4–5 weeks^34^) and peach potato aphid (up to 10 weeks^17^), and demonstrate for the first time that farmers do alter their foliar pesticide application practices as a result of using imidacloprid seed coatings.

Previous work has demonstrated the efficacy of imidacloprid seed coating against peach potato aphids in OSR, however the resulting yield of seed-treated plots was not found to be different from the untreated controls^29^. Indeed, previous studies have failed to explore the yield benefit of neonicotinoid seed coating in comparison to pest control regimes that only use foliar insecticide applications. For the first time we tested whether farmers using neonicotinoid seed treatments increase yield (t ha^−1^) or profit (£ ha^−1^) when compared to OSR crops grown with no seed treatment but with foliar insecticides. Our results demonstrate, that whilst farmers did experience significant yield increases in 2004 and 2006 for imidacloprid and in 2010 for thiamethoxam, there was no consistent effect of imidacloprid seed coating on yield over all years (Fig. 5). Indeed the effects of imidacloprid seed treatment on the yield of oilseed rape were negative in 2008, perhaps suggesting reduced pest control compared to crops that received no insecticide seed treatment. This curious result could be explained by a failure of the prophylactic neonicotinoid seed treatment to control pests, perhaps due to leaching after unprecedented heavy rainfall across many parts of the UK during late summer 2007^35^. Thiamethoxam has been shown to leach from soil with simulated high rainfall^36^ and increased irrigation of seed treated potatoes was shown to reduce imidacloprid residual levels in leaf tissues^37^. It therefore seems plausible that the efficacy of imidacloprid seed treatments may have been compromised in 2008. Interestingly, significant reductions in autumnal foliar insecticide sprays in crops treated with neonicotinoid seed coatings did not appear to greatly influence farming profit, which instead appeared to be driven by OSR yield (compare Figs 5 and 6).

We also present the first evidence of a relationship between increasing imidacloprid usage on OSR seed and escalating honey bee colony losses at a landscape level. Given the generalised nature of our datasets and the multitude of unaccounted variables that are known to impact honey bee colony mortality^38^, it is surprising that our approach had the power to detect a link between imidacloprid usage and honey bee colony loss. Our data were not derived from a controlled experiment and so may be influenced by confounding factors not accounted for in our models. However, controlled studies have severe logistical constraints because the required manipulations focus at the landscape scale^39^. For example, honey bees have been observed flying between control and neonicotinoid treated sites over 10 km apart in agricultural landscapes^40^, placing significant practical restrictions on site selection. Our observational data include colony health measures from 126,220 colonies over 11 years and predicted differences in colony mortality of 10% between low and high field exposure of imidacloprid (Fig. 3; Table 2). Our data would support the suggestion of Cresswell^41^ that published experiments attempting to link neonicotinoid usage with poor honey bee health^40^,^42^ lack the statistical power to discover similar population level effects on colony mortality. Our observational data inform Hill’s epidemiological criteria^43^ of consistency (repeatable association between the putative cause and its consequence over space and time) and provide evidence of a biological gradient^44^. Taken together with a growing body of evidence that imidacloprid alters the foraging behaviour and survival^23^,^24^ of insects, we provide important new supporting evidence to investigate causal relationships between imidacloprid usage at the landscape level and honey bee decline.

The most likely mechanism for our observation is the direct exposure of honey bees after foraging on the nectar and pollen from treated OSR. Nectar taken directly from treated OSR have been shown to contain imidacloprid in the range of 0.6–2.0 ppb^20^ and pollen from honey bees in the USA has been shown to have near ppm levels^8^. Furthermore, feeding choice experiments recently suggested that honey bees may prefer to consume sucrose solutions containing nectar-realistic concentrations of imidacloprid, leading to concerns that preferential foraging on toxin containing nectar could lead to higher colony exposure^45^. Estimated dissipation times (DT_50_) for imidacloprid in soil vary widely^13^ making it difficult to predict whether repeated applications in successive years could result in an accumulation of product in soil. Nevertheless, typically more than 90% of the seed coating enters the soil^13^ and an analyses of soil from UK field detected imidacloprid up to 10.7 ppb, despite no record of growing imidacloprid treated crops for three years prior to sampling^46^. Pollinator-friendly flowering plants growing in field margins of neonicotinoid treated crops have also been shown to contain 1–10 ppb levels in flowers^47^, suggesting another likely route of oral exposure.

More recently imidacloprid usage has declined (Fig. 2), replaced by second generation neonicotinoids with different metabolic fates in honey bees. Whilst clothianidin, thiamethoxam and imidacloprid are all toxic to honey bees, metabolites of imidacloprid, such as olefin, have higher toxicity than the parent compound and the timing of their production has been linked to honey bee mortality^48^. Toxic metabolites of clothianidin and thiamethoxam have not been reported in honey bees (with the exception of conversion of thiamethoxam to clothianidin^49^) and such differences mean that further investigations are required to determine whether the risks for second generation neonicotinoids are significantly different to imidacloprid – parity of risk cannot be assumed. The fact that summed usage of clothianidin, imidacloprid and thiamethoxam failed to explain additional variation in honey bee colony loss could be an artefact of a single year’s sampling (2010; Fig. 2), and this analysis would benefit from the inclusion of more recent usage data. Our model suggested that honey bee colony losses differed significantly between regions and were linked negatively to changes in summer sunshine and spring temperature, but positively to summer temperature. Honey bee colony losses are known to differ between regions in a single year^50^, however our data suggest certain regions, such as Wales, have consistently high losses over time (Tables 1 and 2). Temperature and light intensity have a clear impact on the ability of honey bees to forage and sexually reproduce. Worker honey bees do not begin to forage for pollen and nectar in the spring until temperatures rise above 9 °C, and foraging activity increases as temperature and light intensity rises^51^. Drone (male) honey bees are less likely to depart on mating flights with low light intensity^52^ and honey bee queens mated in poor weather mate with fewer drones^53^ and are more frequently superseded^54^. Reports on weather influencing honey bee colony losses are rare, but colony losses have been associated with unseasonably cool and wet weather in early spring^55^, supporting our observed link between low spring temperatures and honey bee colony losses (Table 1).

For the first time we are able to present the costs and benefits of prophylactic neonicotinoid use on OSR for the farmer alongside an accompanying link to landscape level honey bee colony loss. Neonicotinoid seed coatings provide only partial control of pests and viruses^56^ and resistance in some pest groups^57^ will reduce their efficacy. Risk assessments assuming total control in the presence of a seed coating versus apocalyptic yield losses in their absence are simplistic and perhaps over-state the benefits^32^,^58^. Our data provide numerical evidence on the potential costs (lost honey bee colonies and sometimes lower yield) relative to benefits (reduced number of foliar insecticide sprays and sometimes yield increase) associated with using neonicotinoid seed coatings on OSR. It appears that the economic justification for using neonicotinoid to treat OSR seed in our model system is dynamic and sometimes financially beneficial to farmers. Our data contribute to the growing body of evidence highlighting the need for a large scale field-based experiment to determine the real-world impacts on pollinators of the use of neonicotinoid seed coatings on mass flowering crops. As long as field-applied acute toxins remain the basis of agricultural pest control practices, society will repeatedly be forced to weigh the benefits of pesticides against their collateral environmental damage. Nowhere is this tension more evident than in the system we describe here with the world’s most widely used insecticide, the world’s most widely used managed pollinator and Europe’s most widely grown mass flowering crop.

## Methods

### Exploring factors to explain honey bee colony losses

Pesticide usage surveys of arable crops were completed biennially between 2000 and 2010 by visiting selected holdings stratified from across nine regions of England and Wales (East Midlands, Eastern, London & South East, North East, North West, South West, Wales, West Midlands, Yorkshire & the Humber)^59^. Total neonicotinoid usage on OSR seed (kg/m^2^) was calculated by summing clothianidin, imidacloprid and thiamethoxam seed treatments from national pesticide usage surveys^59^. Regional cropped OSR area and OSR density (m^2^/m^2^) were derived from the June Agricultural Survey biennially between 2000 and 2010^60^. National Bee Unit (NBU) Inspectors assessed the health of honey bee colonies belonging to registered beekeepers across England and Wales between April and September from 2000 to 2010. NBU inspectors operate a combined prioritised, non-random, risk-based inspection protocol for the control of notifiable brood diseases and surveillance for exotic pest incursions^61^. Whilst surveillance visits for exotic pests comprise a random element, apiary visits for notifiable disease can be spatially clustered and diseased colonies are sometimes destroyed. As such, visits where notifiable brood diseases (American or European foulbrood) were discovered were removed from the dataset (approximately 8% of observations). Honey bee hives that contained no live adult honey bees upon inspection were classed as dead colonies. A range of different factors can influence honey bee colony losses, including pests and pathogens, for a recent review see^62^. Whilst the ectoparasitic mite *Varroa destructor* and its associated viruses have been shown to cause honey bee colony losses (for review see^63^), no records of these or any other honey bee pests and diseases were gathered systematically for the period of interest. Honey bee colony losses can be influenced by poor weather conditions during foraging periods^55^, we therefore purchased quarterly meteorological data that covered the majority of the foraging period for honey bees from the Met Office archive of gridded climate data created by interpolating monthly station data as described previously^64^. Measurements of meteorological conditions included average daily mean (*Temp*_*mean*_), minimum (*Temp*_*min*_) and maximum (*Temp*_*max*_) temperature (°C), total rainfall (*Rain*; mm); and total sunshine (*Sun*; h) for Spring (Apr, May, Jun) and Summer (Jul, Aug, Sep) for each region/year combination.

A quasi-binomial generalized linear model with a *logit* link function was constructed using the proportion of honey bee colony losses as the response variable, and region, meteorological conditions (*SpringTemp*_*max*_, *SpringTemp*_*mean*_, *SpringTemp*_*min*_, *SpringSun, SpringRain, SummerTemp*_*max*_, *SummerTemp*_*mean*_, *SummerTemp*_*min*_, *SummerSun, SummerRain*), neonicotinoid usage on OSR seed (kg/m^2^) and density of OSR grown (m^2^/m^2^) as the explanatory variables using Genstat V17.1 (VSN International).

### Quantifying foliar insecticide sprays

The number of foliar insecticide spray applications and active ingredients for winter sown OSR crops were derived from biennial pesticide usage surveys from 2000 to 2010. Timing of insecticide spray application was separated into Autumn (Sept-Nov), and Flowering (Mar-Jul). The number of seasonally applied foliar insecticide sprays (either autumn or during flowering) was used as the response variable in a Poisson regression model with a *log* link function with insecticide seed coating (none, clothianidin, imidacloprid or thiamethoxam) as an explanatory variable and region as a fixed effect. In addition, a mixed effect Poisson regression model was constructed based on data from all years for imidacloprid as a single explanatory variable. The mixed model included a random intercept as well as a random treatment effect grouped by year in order to account for variation in foliar insecticide applications between years. Results were expressed as the effect size between neonicotinoid seed treatment and seed receiving no insecticide seed treatment. Analyses were run using R version 3.0.2 for Windows^61^ and mixed effect models fitted using the *lme4* package for R version 1.0.7^62^.

### Yield benefits of neonicotinoid seed coatings

Yields (t ha^−1^) were derived directly from a random subset of farmers during pesticide usage surveys. Linear models (for each individual year) and mixed effect linear models (imidacloprid use across all years) were constructed in the same way as described above for the number of foliar insecticide sprays, except that the proportion of farm-saved seed and the proportion of hybrid seed were added as additional fixed effects.

### Partial budget analysis of neonicotinoid seed coatings

A partial budget analysis was conducted by multiplying the yield (t ha^−1^) by the fixed sales price of £327.13 t using the July 2007 to mid-April 2013 average delivered Erith OSR price^32^. The costs of applying neonicotinoid seed coatings was subtracted assuming treatment costs of £16.50, £12.90 and £9.55 per ha respectively for farm-saved, conventional and hybrid seed^32^. The cost of applying an exemplar foliar insecticide (deltamethrin) was taken to be £4.75 per spray per ha^32^. Fuel and time costs were assumed to be fixed due to the normal practice of using tank mixes that contain herbicides and/or fungicides when applying foliar insecticides^32^. Linear models (for each individual year) and a mixed effect linear model (imidacloprid use across all years) were constructed in the same way as described above for yield.

## Additional Information

**How to cite this article**: Budge, G.E. *et al*. Evidence for pollinator cost and farming benefits of neonicotinoid seed coatings on oilseed rape. *Sci. Rep*. **5**, 12574; doi: 10.1038/srep12574 (2015).

## Supplementary Material

Supplementary Information

## Figures and Tables

**Figure 1:**
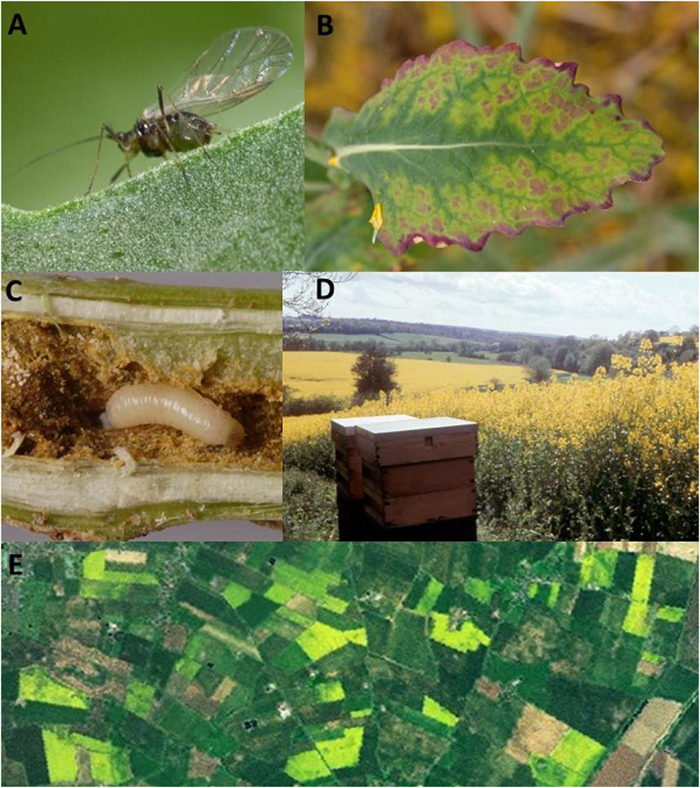
Neonicotinoid insecticides are widely used on oilseed rape in the United Kingdom to combat pests like the peach potato aphid (*Myzus persicae*; **A**), which can carry damaging viruses such as *Turnip yellows virus* (TuYV; **B**), and cabbage stem flea beetle (*Psylliodes chrysocephala*) whose larvae cause stem damage (**C**). Oilseed rape represents a mass flowering nectar and pollen resource in the UK landscape for important pollinators such as the honey bee (**D** and **E**). Images **A**, **C** and **D** – Courtesy Food and Environment Research Agency; Image **B** – Courtesy Dr Mark Stevens, Rothamsted Research; Image **E** created using ESRI ArcGIS 10.1 from the ArcGIS Online World Imagery service.

**Table 1:** Estimated parameters and analysis of deviance for the final quasi-binomial generalized linear model of region, meteorological conditions (average minimum daily summer temperature [°C], cumulative summer sunshine [h], and average maximum spring temperature [°C] for the proportion of dead honey bee colonies from 2000 to 2010).

Parameter	Estimate	s.e.	anti-log of estimate	Df	Deviance ratio	F pr.
Constant Region	−1.96	2.110	0.141			
(*Baseline: North-East*)				8	14.74	<.001
North-West	−0.08	0.613	0.922			
Yorkshire & Humber	−0.51	0.679	0.600			
East Midlands	0.90	0.674	2.464			
West Midlands	0.65	0.709	1.914			
Eastern	0.68	0.710	1.964			
London & SE	0.10	0.734	1.102			
South-West	0.28	0.645	1.319			
Wales	1.27	0.577	3.571			
Meteorological conditions						
*SummerTemp*_*min*_	1.10	0.202	3.006	1	10.64	0.002
*SummerSun*	−0.006	0.0017	0.994	1	5.74	0.021
*SpringTemp*_*max*_	−0.659	0.1790	0.517	1	13.77	<.001
Residual				40		
Total				51		

**Table 2:** Estimated parameters and analysis of deviance for the final quasi-binomial generalized linear model of region and imidacloprid usage on OSR seed (kg/m^2^) for the proportion of dead honey bee colonies from 2000 to 2010.

Parameter	Estimate	s.e.	anti-log of estimate	Df	Deviance ratio	F pr.
Constant Region	−3.30	0.622	0.037			
(*Baseline: North-East*)				8	11.26	<.001
North-West	0.76	0.668	2.144			
Yorkshire & Humber	−0.41	0.734	0.667			
East Midlands	0.43	0.658	1.535			
West Midlands	0.11	0.683	1.111			
Eastern	0.39	0.640	1.475			
London & SE	−0.37	0.650	0.688			
South-West	0.46	0.636	1.587			
Wales	1.87	0.632	6.479			
Imidacloprid usage	19.3	5.76	2.4E+08	1	11.61	0.001
Residual				42		
Total				51		

**Table 3:** Sample size and planted area of OSR (ha) used to assess the number of foliar insecticide sprays, yield and economic analysis for crops that received no insecticide seed treatment (none) or neonicotinoid seed treatments (clothianidin, imidacloprid or thiamethoxam).

Year	None		Imidacloprid		Clothianidin		Thiamethoxam	
	No. fields	Area (ha)	No. fields	Area (ha)	No. fields	Area (ha)	No. fields	Area (ha)
*Foliar insecticide sprays*								
2002	400	6,604	253	4,913	—	—	—	—
2004	305	5,121	522	8,395	—	—	—	—
2006	267	4,782	716	10,473	—	—	—	—
2008	173	3,631	814	14,077	—	—	—	—
2010	50	1,204	150	3,065	244	4,241	336	6,547
*Yield and economic analysis*								
2002	179	2,674	95	1,720	—	—	—	—
2004	167	2,846	311	4,986	—	—	—	—
2006	190	2,832	585	8,452	—	—	—	—
2008	125	2,190	614	11,204	—	—	—	—
2010	45	1,091	92	1,607	192	3,366	206	4,152

## References

[b1] Ollerton J., Winfree R. & Tarrant S. How many flowering plants are pollinated by animals? Oikos 120, 321–326 (2011).

[b2] Eilers E. J., Kremen C., Greenleaf S. S., Garber A. K. & Klein A. M. Contribution of Pollinator-Mediated Crops to Nutrients in the Human Food Supply. Plos One 6, 10.1371/journal.pone.0021363 (2011). PMC3120884 21731717

[b3] Aizen M. A. & Harder L. D. The Global Stock of Domesticated Honey Bees Is Growing Slower Than Agricultural Demand for Pollination. Curr Biol 19, 915–918 (2009). 19427214 10.1016/j.cub.2009.03.071

[b4] Forister M. L. . Compounded effects of climate change and habitat alteration shift patterns of butterfly diversity. PNAS 107, 2088–2092 (2010). 20133854 10.1073/pnas.0909686107 PMC2836664

[b5] Biesmeijer J. . Parallel declines in pollinators and insect-pollinated plants in Britain and the Netherlands. Science 313, 351–354 (2006). 16857940 10.1126/science.1127863

[b6] Garratt M. . The identity of crop pollinators helps target conservation for improved ecosystem services. Biol Conserv 169, 128–135 (2014). 24696525 10.1016/j.biocon.2013.11.001 PMC3969722

[b7] Barr C. J. . Countryside Survey 1990: Main Report (Countryside 1990 vol. 2). (London, 1993).

[b8] Mullin C. A. . High Levels of Miticides and Agrochemicals in North American Apiaries: Implications for Honey Bee Health. Plos One 5, 10.1371/journal.pone.0009754 (2010). PMC2841636 20333298

[b9] Bingham G. . Temporal synergism can enhance carbamate and neonicotinoid insecticidal activity against resistant crop pests. Pest Manag Sci 64, 81–85 (2008). 17926308 10.1002/ps.1477

[b10] Tomizawa M. & Casida J. E. Neonicotinoid insecticide toxicology: Mechanisms of selective action. Annu Rev Pharmacol 45, 247–268 (2005). 10.1146/annurev.pharmtox.45.120403.095930 15822177

[b11] Laurino D., Porporato M., Patetta A. & Manino A. Toxicity of neonicotinoid insecticides to honey bees: laboratory tests. Bull Insectology 64, 107–113 (2011).

[b12] Jeschke P., Nauen R., Schindler M. & Elbert A. Overview of the Status and Global Strategy for Neonicotinoids. J Agr Food Chem 59, 2897–2908 (2011). 20565065 10.1021/jf101303g

[b13] Goulson D. REVIEW: An overview of the environmental risks posed by neonicotinoid insecticides. J Appl Ecol 50, 977–987 (2013).

[b14] Carre P. & Pouzet A. Rapeseed market, worldwide and in Europe. OCL - Oilseeds and Fats, Crops and Lipids 21, D102–D102 (2014).

[b15] Hayter K. E. & Cresswell J. E. The influence of pollinator abundance on the dynamics and efficiency of pollination in agricultural Brassica napus: implications for landscape-scale gene dispersal. J Appl Ecol 43, 1196–1202 (2006).

[b16] Westphal C., Steffan-Dewenter I. & Tscharntke T. Mass flowering crops enhance pollinator densities at a landscape scale. Ecol Lett 6, 961–965 (2003).

[b17] Jones R., Coutts B. & Hawkes J. Yield-limiting potential of Beet western yellows virus in *Brassica napus*. Aust J Agr Res 58, 788–801 (2007).

[b18] Bass C. . The evolution of insecticide resistance in the peach potato aphid, Myzus persicae. Insect Biochem Molec 51, 41–51 (2014). 10.1016/j.ibmb.2014.05.003 24855024

[b19] Zimmer C. T., Mueller A., Heimbach U. & Nauen R. Target-site resistance to pyrethroid insecticides in German populations of the cabbage stem flea beetle, Psylliodes chrysocephala L. (Coleoptera: Chrysomelidae). Pestic Biochem Phys 108, 1–7 (2014). 10.1016/j.pestbp.2013.11.005 24485308

[b20] Pohorecka K. . Residues of Neonicotinoid Insecticides in Bee Collected Plant Materials from Oilseed Rape Crops and Their Effect on Bee Colonies. J Apic Sci 56, 115–134 (2012).

[b21] EFSA. Statement on the findings in recent studies investigating sub-lethal effects in bees of some neonicotinoids in consideration of the uses currently authorised in Europe. EFSA Journal 10, 2752 (2012).

[b22] Feltham H., Park K. & Goulson D. Field realistic doses of pesticide imidacloprid reduce bumblebee pollen foraging efficiency. Ecotoxicology 23, 317–323 (2014). 24448674 10.1007/s10646-014-1189-7

[b23] Schneider C. W., Tautz J., Gruenewald B. & Fuchs S. RFID Tracking of Sublethal Effects of Two Neonicotinoid Insecticides on the Foraging Behavior of *Apis mellifera*. Plos One 7, 10.1371/journal.pone.0030023 (2012). PMC3256199 22253863

[b24] Henry M. . A Common Pesticide Decreases Foraging Success and Survival in Honey Bees. Science 336, 348–350 (2012). 22461498 10.1126/science.1215039

[b25] Whitehorn P. R., O’Connor S., Wackers F. L. & Goulson D. Neonicotinoid Pesticide Reduces Bumble Bee Colony Growth and Queen Production. Science 336, 351–352 (2012). 22461500 10.1126/science.1215025

[b26] Sandrock C. . Impact of Chronic Neonicotinoid Exposure on Honeybee Colony Performance and Queen Supersedure. Plos One 9, 10.1371/journal.pone.0103592 (2014). PMC4118897 25084279

[b27] Gill R. J., Ramos-Rodriguez O. & Raine N. E. Combined pesticide exposure severely affects individual- and colony-level traits in bees. Nature 491, 105–108 (2012). 23086150 10.1038/nature11585 PMC3495159

[b28] EU. EU-485/2013: Amending implementing regulation (EU) No 540/2011. http://eur-lex.europa.eu/legal-content/EN/TXT/?uri=CELEX:32013R0485 (2013) (Date of access: 04/11/2014).

[b29] Dewar A., Tait M. & Stevens M. Efficacy of thiamethoxam seed treatment against aphids and turnip yellows virus in oilseed rape. Asp Appl Biol, 195–202 (2011).

[b30] Soroka J. J., Grenkow L. F. & Irvine R. B. Impact of decreasing ratios of insecticide-treated seed on flea beetle (Coleoptera: Chrysomelidae, Phyllotreta spp.) feeding levels and canola seed yields. J Econ Entomol 101, 1811–1820 (2008). 19133461 10.1603/0022-0493-101.6.1811

[b31] Birch P. A. & Nicholson T. A new insecticidal seed treatment for oilseed rape. In: Seed treatment: challenges & opportunities, British Crop Protection Council, 27–32 (2001).

[b32] Nicholls C. J. Implications of the restriction on the neonicotinoids: imidacloprid, clothianidin and thiamethoxam on crop protection in oilseeds and cereals in the UK. Research Review No. 77. (2013).

[b33] Allsopp M. H., de Lange W. J. & Veldtman R. Valuing Insect Pollination Services with Cost of Replacement. Plos One 3, 10.1371/journal.pone.0003128 (2008). PMC2519790 18781196

[b34] Kazda J., Baranyk P. & Nerad D. The implication of seed treatment of winter oilseed rape. Plant Soil Environ 51, 403–409 (2005).

[b35] Prior J. & Beswick M. The exceptional rainfall of 20 July 2007. Weather 63, 261–267, 10.1002/wea.308 (2008).

[b36] Gupta S., Gajbhiye V. T. & Gupta R. K. Soil dissipation and leaching behavior of a neonicotinoid insecticide thiamethoxam. Bul Environ Contam Tox 80, 431–437, 10.1007/s00128-008-9420-y (2008). 18431522

[b37] Prager S. M., Vindiola B., Kund G. S., Byrne F. J. & Trumble J. T. Considerations for the use of neonicotinoid pesticides in management of Bactericera cockerelli (Sulk) (Hemiptera: Triozidae). Crop Prot 54, 84–91, 10.1016/j.cropro.2013.08.001 (2013).

[b38] vanEngelsdorp D. . A national survey of managed honey bee 2010-11 winter colony losses in the USA: results from the Bee Informed Partnership. J Apicult Res 51, 115–124 (2012).

[b39] vanEngelsdorp D. . Standard epidemiological methods to understand and improve Apis mellifera health. J Apicult Res 52, 10.3896/ibra.1.52.4.15 (2013).

[b40] Cutler G. C., Scott-Dupree C. D., Sultan M., McFarlane A. D. & Brewer L. A large-scale field study examining effects of exposure to clothianidin seed-treated canola on honey bee colony health, development, and overwintering success. Peer J 2, e652–e652 (2014). 25374790 10.7717/peerj.652 PMC4217196

[b41] Cresswell J. E. A meta-analysis of experiments testing the effects of a neonicotinoid insecticide (imidacloprid) on honey bees. Ecotoxicology 20, 149–157 (2011). 21080222 10.1007/s10646-010-0566-0

[b42] Pilling E., Campbell P., Coulson M., Ruddle N. & Tornier I. A Four-Year Field Program Investigating Long-Term Effects of Repeated Exposure of Honey Bee Colonies to Flowering Crops Treated with Thiamethoxam. Plos One 8, 10.1371/journal.pone.0077193 (2013). PMC3806756 24194871

[b43] Hill A. B. The Environment and disease: Association or Causation? P Roy Soc Med 58, 295–300 (1965). 10.1177/003591576505800503 PMC1898525 14283879

[b44] Cresswell J. E., Desneux N. & vanEngelsdorp D. Dietary traces of neonicotinoid pesticides as a cause of population declines in honey bees: an evaluation by Hill’s epidemiological criteria. Pest Manag Sci 68, 819–827 (2012). 22488890 10.1002/ps.3290

[b45] Kessler S. C. . Bees prefer foods containing neonicotinoid pesticides. Nature 521, 74–76 (2015). 25901684 10.1038/nature14414 PMC4772122

[b46] Jones A., Harrington P. & Turnbull G. Neonicotinoid concentrations in arable soils after seed treatment applications in preceding years. Pest Manag Sci 70, 1780–1784, 10.1002/ps.3836 (2014). 24888990

[b47] Krupke C. H., Hunt G. J., Eitzer B. D., Andino G. & Given K. Multiple Routes of Pesticide Exposure for Honey Bees Living Near Agricultural Fields. Plos One 7, 10.1371/journal.pone.0029268 (2012). PMC3250423 22235278

[b48] Suchail S., Debrauwer L. & Belzunces L. P. Metabolism of imidacloprid in Apis mellifera. Pest Manag Sci 60, 291–296 (2004). 15025241 10.1002/ps.772

[b49] Nauen R., Ebbinghaus-Kintscher U., Salgado V. L. & Kaussmann M. Thiamethoxam is a neonicotinoid precursor converted to clothianidin in insects and plants. Pestic Biochem Phys 76, 55–69 (2003).

[b50] Steinhauer N. A. . A national survey of managed honey bee 2012-2013 annual colony losses in the USA: results from the Bee Informed Partnership. J Apicult Res 53, 1–18, 10.3896/ibra.1.53.1.01 (2014).

[b51] Burrill R. M. & Dietz A. The response of honey bees to variations in solar-radiation and temperature. Apidologie 12, 319–328, 10.1051/apido:19810402 (1981).

[b52] Neves E. F., Faita M. R., Gaia L. D. O., Alves Junior V. V. & Antonialli-Junior W. F. Influence of Climate Factors on Flight Activity of Drones of Apis mellifera (Hymenoptera: Apidae). Sociobiology 57, 107–113 (2011).

[b53] Woyke J. Causes of repeated mating flights by queen honeybees. J Apicult Res 3, 7 (1964).

[b54] Winston M. The biology of the honey bee. (Harvard University Press, 1991).

[b55] Wilson W. & Menapace D. Disappearing disease of honey bees: a survey of the United States. Am Bee J 119, 118–217 (1979).

[b56] Coutts B., Webster C. & Jones R. Control of Beet western yellows virus in *Brassica napus* crops: infection resistance in Australian genotypes and effectiveness of imidacloprid seed dressing. Crop Pasture Sci 61, 321–330 (2010).

[b57] Puinean A. M. . Amplification of a Cytochrome P450 Gene Is Associated with Resistance to Neonicotinoid Insecticides in the Aphid Myzus persicae. Plos Genetics 6, 10.1371/journal.pgen.1000999 (2010). PMC2891718 20585623

[b58] Noleppa S. & Hahn T. The value of neonicotinoid seed treatment in the European Union: a socioeconomic, technological and environmental review, HFFA working paper 01/2013 (2013).

[b59] Garthwaite D. G., Thomas M. R., Parrish G., Smith L. & Barker I. Pesticide usage survey report 224: Arable crops in Great Britain. (2008).

[b60] Defra. June Census of Agriculture and Horticulture (Land Use and Livestock on Agricultural Holdings at 1 June 2010) UK - Final Results. (2010) (Date of access: 01/02/2012).

[b61] Wilkins S., Brown M. A. & Cuthbertson A. G. S. The incidence of honey bee pests and diseases in England and Wales. Pest Manag Sci 63, 1062–1068 (2007). 17879983 10.1002/ps.1461

[b62] Vanbergen A. J. & Initiative T. I. P. Threats to an ecosystem service: pressures on pollinators. Front Ecol Environ 11, 251–259 (2013).

[b63] Rosenkranz P., Aumeier P. & Ziegelmann B. Biology and control of Varroa destructor. J invertebr pathol 103 Suppl 1, S96–119, 10.1016/j.jip.2009.07.016 (2010). 19909970

[b64] Perry M. & Hollis D. The generation of monthly gridded datasets for a range of climatic variables over the UK. Int J Climatol 25, 1041–1054 (2005).

