# Supplementary material for: Evidence for pollinator cost and farming benefits of neonicotinoid seed coatings on oilseed rape
Source: Sci Rep. 2015 Aug 13;5:12574. doi: 10.1038/srep12574 (PMC4535276; doi:10.1038/srep12574)
Supplement: Supplementary Information [file srep12574-s1.pdf]

Supplementary Information

Evidence for pollinator cost and farming benefits of neonicotinoid seed coatings on oilseed rape

Giles E. Budge, Dave Garthwaite, Andrew Crowe, Nigel D. Boatman, Keith S. Delaplane, Mike A. Brown, Helene H. Thygesen and Stéphane Pietravalle

Supplementary Table S1 Regional data used to analyse honey bee colony losses

Region	Year	# honey bee colonies inspected[i]	# honey bee colonies found dead[i]	Density of oilseed rape grown (m^2/m^2)[ii]	Neonicotinoid usage on oilseed rape (kg/m^2)[iii]	Imidacloprid usage on oilseed rape (kg/m^2)[iii]	Spring Temp $_{max}$ (°C)	Spring Temp $_{mean}$ (°C)	Spring Temp $_{min}$ (°C)	Spring Sunshine (h)	Spring Rainfall (mm)	Summer $Temp_{max}$ (°C)	Summer $Temp_{mean}$ (°C)	Summer $Temp_{min}$ (°C)	Summer Sunshine (h)	Summer Rainfall (mm)
North-East	2000	687	22	1.62E-02	0	0	13.42	9.34	5.33	466.5	273.7	17.32	13.36	9.45	401.6	254.8
North-West	2000	1304	96	1.88E-03	0	0	14.67	10.57	6.45	525.8	259.0	18.10	14.32	10.54	447.0	327.2
Yorkshire & Humber	2000	521	39	2.34E-02	4.76E-10	4.76E-10	14.72	10.61	6.52	471.5	275.3	18.55	14.52	10.50	411.7	238.9
East Midlands	2000	1358	46	4.49E-02	1.20E-09	1.20E-09	15.77	11.41	7.05	475.1	237.7	19.81	15.35	10.89	441.0	202.8
Eastern	2000	2677	227	3.36E-02	0	0	16.51	12.16	7.82	516.0	202.9	20.59	16.12	11.63	473.6	180.4
London & SE	2000	3467	120	2.60E-02	0	0	16.37	12.10	7.84	500.1	256.2	20.61	16.10	11.60	505.3	202.1
South-West	2000	3710	209	1.05E-02	0	0	15.43	11.43	7.45	494.2	285.9	19.75	15.65	11.57	502.2	267.9
Wales	2000	3163	605	7.99E-04	0	0	14.60	10.50	6.49	502.7	325.6	18.41	14.46	10.60	458.6	350.2
North-East	2002	406	9	2.07E-02	1.16E-08	1.16E-08	14.00	10.03	6.06	484.9	175.0	17.82	13.87	9.84	412.7	200.4
North-West	2002	1487	109	2.06E-03	0	0	14.72	10.83	7.00	494.7	291.5	18.20	14.42	10.67	426.4	253.7
Yorkshire & Humber	2002	322	15	2.65E-02	1.41E-08	1.41E-08	15.40	11.11	6.82	517.5	166.4	19.10	14.95	10.80	413.7	240.9
East Midlands	2002	747	24	5.14E-02	1.76E-08	1.76E-08	16.43	11.75	7.08	542.9	132.2	20.36	15.75	11.12	459.6	209.0
West Midlands	2002	1490	136	1.77E-02	6.85E-09	6.85E-09	16.07	11.52	6.99	539.4	162.0	20.05	15.43	10.80	481.8	153.4
Eastern	2002	3449	141	3.82E-02	1.12E-08	1.12E-08	16.93	12.28	7.66	568.9	124.2	21.18	16.51	11.86	490.1	155.4
London & SE	2002	3763	121	2.67E-02	1.41E-08	1.41E-08	16.66	12.20	7.76	590.5	183.8	21.04	16.33	11.62	551.5	156.7
South-West	2002	3703	168	1.12E-02	6.96E-09	6.96E-09	15.44	11.43	7.43	547.5	233.9	19.77	15.48	11.21	536.1	152.5
Wales	2002	2698	473	4.95E-04	0	0	14.37	10.54	6.81	477.4	323.2	18.26	14.21	10.23	446.6	185.3
North-East	2004	496	49	2.87E-02	2.51E-08	2.51E-08	14.68	10.53	6.38	484.0	192.8	17.77	13.95	10.07	454.7	308.2
North-West	2004	1694	116	2.08E-03	1.55E-09	1.55E-09	15.23	11.30	7.41	493.5	207.6	18.31	14.75	11.20	450.6	435.1
Yorkshire & Humber	2004	530	5	4.17E-02	2.91E-08	2.91E-08	15.81	11.53	7.24	488.2	200.6	19.06	15.05	11.05	470.1	294.7
East Midlands	2004	1606	66	6.70E-02	3.95E-08	3.95E-08	16.90	12.26	7.63	515.9	175.6	20.40	15.95	11.47	498.2	276.4
West Midlands	2004	1977	46	2.75E-02	2.53E-08	2.53E-08	16.78	12.19	7.62	530.4	179.1	20.09	15.72	11.34	487.4	243.6
Eastern	2004	2972	172	4.63E-02	3.68E-08	3.68E-08	17.36	12.63	7.90	539.8	142.7	21.43	16.79	12.17	536.7	217.1
London & SE	2004	3592	128	3.89E-02	3.51E-08	3.51E-08	17.45	12.67	7.89	587.4	157.9	21.36	16.77	12.17	562.6	191.8
South-West	2004	3723	340	1.95E-02	1.72E-08	1.72E-08	16.65	12.25	7.89	607.2	176.7	19.91	15.89	11.88	514.6	245.4
Wales	2004	3958	870	1.40E-03	9.63E-10	9.63E-10	15.30	11.25	7.29	510.4	221.0	18.39	14.77	11.19	443.1	379.8
North-East	2006	344	5	2.56E-02	3.16E-08	3.16E-08	14.57	10.08	5.61	597.5	153.6	20.01	15.47	10.90	593.4	193.7

North-West	2006	1741	157	2.22E-03	1.46E-09	1.46E-09	15.27	11.12	7.04	545.6	237.2	20.40	16.15	11.92	562.4	270.7
Yorkshire & Humber	2006	1291	52	3.92E-02	3.86E-08	3.86E-08	15.69	11.23	6.76	551.0	176.9	21.18	16.58	11.96	579.4	238.9
East Midlands	2006	556	24	7.18E-02	5.68E-08	5.68E-08	16.76	12.14	7.53	527.2	144.6	22.50	17.53	12.55	607.5	212.4
West Midlands	2006	1349	158	2.65E-02	2.68E-08	2.68E-08	16.86	12.24	7.63	548.5	163.0	22.25	17.29	12.31	582.6	184.6
Eastern	2006	3779	563	5.29E-02	5.18E-08	5.18E-08	17.22	12.50	7.80	553.5	135.2	23.22	18.24	13.29	609.8	193.7
London & SE	2006	4605	256	3.76E-02	3.27E-08	3.27E-08	17.38	12.71	8.06	591.4	150.7	23.15	18.28	13.40	653.6	174.5
South-West	2006	3738	357	1.84E-02	1.75E-08	1.75E-08	16.46	12.18	7.90	598.5	180.1	21.80	17.38	12.97	615.5	179.0
North-East	2008	183	25	3.05E-02	2.87E-08	2.74E-08	13.87	9.77	5.69	508.4	202.3	17.50	13.95	10.38	358.1	408.7
North-West	2008	1508	203	2.57E-03	2.44E-09	2.44E-09	14.98	10.88	6.83	497.6	211.5	17.76	14.41	11.08	343.9	422.9
Yorkshire & Humber	2008	1406	82	4.92E-02	3.08E-08	3.08E-08	15.10	10.76	6.42	490.8	184.5	18.74	14.91	11.06	396.8	343.0
East Midlands	2008	2463	609	8.50E-02	4.21E-08	4.21E-08	16.18	11.57	6.99	524.3	152.9	19.87	15.59	11.31	431.6	240.6
West Midlands	2008	1386	80	3.34E-02	2.72E-08	2.72E-08	16.17	11.60	7.07	503.9	169.1	19.24	15.15	11.05	389.7	304.9
Eastern	2008	3551	409	6.65E-02	5.34E-08	5.34E-08	16.88	12.18	7.52	570.7	157.0	20.59	16.17	11.74	464.5	186.6
London & SE	2008	3683	171	4.21E-02	4.36E-08	4.36E-08	17.26	12.55	7.86	602.4	189.9	20.02	15.84	11.68	478.2	218.2
South-West	2008	3960	334	2.01E-02	1.93E-08	1.93E-08	16.30	11.99	7.69	605.5	211.8	18.84	15.12	11.38	435.2	368.0
Wales	2008	4081	1068	1.48E-03	1.78E-09	1.78E-09	15.09	10.95	6.90	539.2	244.4	17.62	14.17	10.79	357.3	491.4
North-East	2010	196	2	2.98E-02	6.26E-08	1.06E-08	14.58	9.94	5.33	563.4	89.0	17.71	13.74	9.79	445.3	248.2
North-West	2010	1836	37	3.49E-03	5.96E-09	7.17E-10	15.34	10.73	6.17	590.0	96.7	17.75	14.23	10.76	395.6	394.3
Yorkshire & Humber	2010	2976	66	5.54E-02	1.11E-07	1.21E-08	15.65	10.87	6.07	585.9	84.1	18.95	14.87	10.78	419.8	222.1
East Midlands	2010	3076	108	9.58E-02	1.38E-07	1.35E-08	16.69	11.63	6.58	642.1	90.3	20.07	15.63	11.18	438.7	210.9
West Midlands	2010	1869	61	3.85E-02	8.31E-08	2.27E-08	16.97	11.69	6.43	641.2	108.4	19.44	15.14	10.86	401.5	207.6
Eastern	2010	4945	452	7.20E-02	1.33E-07	1.69E-08	17.02	11.80	6.60	659.1	71.0	21.10	16.52	11.95	466.8	211.2
London & SE	2010	5996	180	4.04E-02	7.85E-08	1.02E-08	17.36	12.02	6.69	690.9	84.4	20.77	16.22	11.68	474.5	176.9
South-West	2010	5408	252	2.23E-02	2.99E-08	7.19E-09	16.73	11.75	6.78	692.9	102	19.62	15.50	11.39	460.4	251.5
Wales	2010	4794	662	1.60E-03	1.70E-09	1.70E-09	15.65	10.78	5.96	660.0	138.3	17.80	14.20	10.67	397.4	415.0

[i] Data derived from the National Bee Unit inspection programme (www.nationalbeeunit.com)

[ii] Data derived from the June survey data for the respective years

[i] Data derived from the pesticide usage surveys from the respective years
